# Decoupling the use and meaning of strategic plans in public healthcare

**DOI:** 10.1186/1472-6963-13-5

**Published:** 2013-01-04

**Authors:** Federico Lega, Francesco Longo, Andrea Rotolo

**Affiliations:** 1 Department for Institutional Analysis and Public Management, Bocconi University Milan, Via Roentgen 1, 20136, Milan, Italy

**Keywords:** Strategy, Strategic planning, Planning drivers, Public healthcare organisations, Coherence, Consistency, Management tools

## Abstract

**Background:**

The culture of New Public Management has promoted the diffusion of strategic management tools throughout Public Healthcare Organisations (PHOs). There is consensus that better strategic planning tools are required to achieve higher levels of organisational performance. This paper provides evidence and understanding of the emergent uses and scope of strategic planning in PHOs, in order to answer three research questions: (i) has the New Public Management approach changed the organisational culture of PHOs in terms of how they adopt, diffuse, and use strategic planning documents? (ii) how coherent are strategic planning documents in PHOs? and (iii) what are the main purposes of strategic documents in PHOs?

**Methods:**

An analysis was carried out in three Italian Local Health Authorities. We analysed the number and types of formal strategic documents adopted between 2004 and 2012, evaluating their degree of coherence and coordination, their hierarchy, their degree of disclosure, and the consistency of their strategic goals. A content analysis was performed to investigate overlap in terms of content and focus, and a qualitative analysis was carried out to study and represent the relationships between documents.

**Results:**

The analysis showed that a rich set of strategic documents were adopted by each PHO. However, these are often uncoordinated and overlap in terms of content. They adopt different language and formats for various stakeholders. The presence of diverse external drivers may explain the divergent focus, priorities and inconsistent goals in the strategic documents. This planning complexity makes it difficult to determine how the overall goals and mission of an organisation are defined and made visible.

**Conclusions:**

The evidence suggests that PHOs use a considerable number of strategic documents. However, they employ no clear or explicit overarching strategy currently, and strategic planning appears to be externally oriented. All the documents communicate similar topics to different stakeholders, although they use different language to answer to the different expectations of each stakeholder. Therefore, strategic planning and plans seem to be driven by neo-institutional approaches, as they are means to build consensus and negotiate ground for strategic actions, rather than means to identify strategic choices and priorities.

## Background

This paper provides evidence of the manifold uses and scope of strategic planning in Italian public healthcare organisations (PHOs). We analyse the features and scope of formally adopted strategic documents that Italian PHOs are expected to produce, or that they autonomously decide to develop.

There is some consensus in the literature that - especially in turbulent and complex contexts - strategic tools are required to achieve higher levels of performance [1,2]. This view has been strongly influenced and supported by the New Public Management (NPM) scholars. They stress that public sector organisations must adopt strategic planning practices if they are to improve their management in the long-term, to guide public institutions, and to improve their performance in an increasingly turbulent context [3]. An alternative view, put forward by a number of academics and practitioners, is that no clear strategy can apply to public sector organisations because they are political and ethical in nature, and it is, therefore, politicians who must make the most important decisions in this regard [4]. However, the reality of effective management of PHOs lies somewhere between the two viewpoints.

The formulation of strategy in the public sector requires a disciplined effort to take the fundamental decisions and actions that shape and guide what an organisation is, what it does, and why it does it, all within a legal framework [5,6].

It is clear that political discourse often lies behind the success or failure of efforts to strategise in public contexts, in which a number of diverse actors come together to define goals and implement actions. As suggested by the strategy-as-practice approach, the formulation of strategy in PHOs is often the result of the interplay between organisational discourse and politics in the public sector.

In fact, strategic decision processes in PHOs suffer from the effects of the public nature of these organisations, beside the effect of the peculiarities of the healthcare sector. The public nature, according to the published literature, has clear effects on the organisational environment [7,8]:

• Complexity. Public organisations generally have a variety of stakeholders to refer to. Often, the requirements of the various external constituencies (e.g., taxpayers and the recipients of services or industrial groups) are in conflict. Public organisations are expected to contribute to the general development of the community, while simultaneously pursuing their primary goals.

• Permeability. Public organisations are ‘open systems’ that are easily influenced by external events, especially with regard to making services responsive to emergent public needs. This pushes constituents’ daily influence and delays the formulation of long-term policies and the implementation of processes.

• Instability. Political constraints result in frequent policy changes and the imposition of short time horizons on public managers. The political cycle often creates pressure to achieve results rapidly or makes it extremely difficult to pursue medium-term projects that need continuity to be implemented successfully.

Second, the concept of publicness affects the goals of organisations. It has been frequently disputed that public organisations have distinctive goals such as equity and transparency that do not exist in the private sector.

Third, the internal characteristics of public organisations are considered to be distinctive for many different reasons: public sector organisations have more formal procedures for decision making and are less flexible and more risk-averse than their counterparts in the private sector; the existence of weighty procedures and regulations implies a counter-productive obsession with rules and processes, rather than a concern with results and outcomes; managers in public organisations have less freedom to act and react according to their personal views of the circumstances that they face; public managers experience the impact of hierarchy (bureaucratic rules and regulations) without its benefits (the freedom and power to manage their subordinates); and the level of organisational commitment is believed to be lower in the public sector, largely because of weak links between performance and organisational rewards.

The healthcare sector is a complex environment, where strategy and decision-making are defined by the intersection of different cultures (professional, bureaucratic and managerial), values (political, social, entrepreneurial, individual) and interests (different professional families, groups and individuals, firms and the public sector, different levels of government). Furthermore, management in the healthcare sector is becoming increasingly more difficult, as every system is subject to the same structural trends: a demographic shift toward the elderly, the skyrocketing pace of technological innovation and rising consumer expectations, all of which impact heavily on rising costs.

Consequently, strategic documents may not be used as they were originally intended, because they often end up responding more to the constraints of institutional isomorphism than they do to the primary need to formulate strategy.

The backdrop to the present study is more than 15 years of managerialisation in the Italian health service [9], which was strongly influenced by the principles of NPM. The aim of the study was to investigate whether PHOs use strategic documents to support their actual strategy and decision-making processes, or whether these documents serve some of the purposes highlighted by other authors, namely building consensus, advertising, testing internal management competence, and responding isomorphically to institutional requests.

In order to formulate our three research questions, we began by assessing evidence of whether PHOs adopt strategic plans concurrently. We then investigated the following research questions:

1. Has the NPM approach changed the organisational culture of PHOs in terms of how they adopt, diffuse, and use strategic planning documents?

2. How coherent are strategic documents in PHOs (assessing their focuses, language use, and degree of coordination)?

3. What are the main purposes of strategic documents in PHOs?

PHOs are pluralistic organisations with diffuse structures of governance, and the process of agreeing strategic decisions can therefore be difficult. We designed our study to explore the presence, consistency and scope of PHOs’ strategic documents. Our findings are discussed with reference to two theories: the political environment framework and the garbage can framework.

In the political environment framework, the public sector is seen as a number of parallel but separate strategy-making processes that are politically driven, ambiguous in their goals, and not well formalised. Mintzberg [10] argued that planning and the decisions that result from it are biased, and not as rational or apolitical as previous authors have implied. For example, plans and planners inevitably favour quantitative information because it is easier to use and decipher than qualitative information, in contrast to politicians and public policy makers who favour qualitative information. Cohen and March [11] added that strategic plans play no real strategic role, but instead either serve a symbolic purpose, or are useful tools for administrators who require a basis for agreeing to, or challenging, departmental proposals.

The garbage can framework suggests that public organisations lack formal long-term planning processes. Their strategies are instead developed gradually through disjointed incrementalism, learning-by-doing, and the recognition/formalisation of emergent strategies. Moreover, in the real world, decisions, including strategic ones, arise from the accidental or random confluence of four streams [12,13]. These are: (i) choice opportunities (i.e., occasions that call for a decision); (ii) solutions to problems; (iii) participants (i.e., people with busy schedules who might pay attention); and (iv) problems in the form of the concerns of people within and outside the organisation. Thus, according to Eisenhardt and Zbaracki, “decision making occurs in a stochastic meeting of choices looking for problems, problems looking for choices, solutions looking for problems to answer, and decision makers looking for something to decide” [14]. In other words, the garbage can model emphasises the importance of chance and the random nature of events.

Our hypothesis complements the theoretical frameworks presented above. We propose that, in a complex environment characterised by a number of sources of pressure on Italian PHOs, strategic planning documents are often the result of the interplay between key stakeholders, and these documents are internally inconsistent because they are drafted to meet different external expectations. Strategic planning documents can be used to build consensus and could be a means of satisfying the informational needs of different stakeholders. This could create a space for actual strategic decision-making outside the remit of official planning documents.

## Methods

Strategic planning documents can have a bureaucratic origin, since they can represent an administrative constraint imposed by law or by a higher level of government, or they can represent an internal managerial resolution to steer the organisation. External legal constraints do not impose logical links between strategic documents, or the attainment of specific overall goals. Each document can explicitly pursue different objectives, and the differences between documents are often the result of legislative stratification or of a fragmented geography of power in the public network. In light of this framework, the authors focused on understanding the degree of coherence between different documents from the same LHA.

Therefore, the aim of the present study was to answer the three research questions, posed in Section 1, by analysing and understanding the scope and overall degree of coherence of all the strategic documents taken into account.

In order to answer the first question, we analysed a number of different formal strategic documents for each PHO and interviewed their top managers.

In order to answer the second research question, we evaluated the degree of coherence and coordination between strategic tools, or the existence of a clear hierarchy between them, by considering both their timing and their focuses. The underlying assumption is that a high degree of integration and a clear time sequence are indicators of a clear strategy aimed at effective organisational guidelines. By contrast, if the language use in strategic documents is uncoordinated and no overarching strategy can be discerned from them, this implies a weak internal commitment to formalise a strategic design.

In order to answer the third research question, we analysed the degree of disclosure and the consistency of the strategic goals expressed in the different documents. A high level of disclosure and consistency implies that the PHOs concerned intend to share strategic goals with internal and external stakeholders, as well as the actions necessary to implement them. By contrast, if there is no real attempt to satisfy the informational needs of stakeholders or to provide clear and visible results, there is a low level of consistency. The presence of quantitative service targets and future resource allocation is a strong indicator of disclosure, while the presence of only qualitative suggestions for the future can be considered the opposite.

### Sample selection

Data were collected from the Italian SSN (*Sistema Sanitario Nazionale*). The Italian SSN is a Beveridge-like national healthcare system that includes 140 local health authorities (LHAs) that are the basic purchasers and providers of the system, and 80 independent hospital trusts that represent the most specialised third-level providers. PHOs in the SSN have average annual budgets of 480 million Euros, and have an average of 3 000 employees. In Italy (population 60 million), the SSN is managed by 21 regional governments. The LHAs in each region show different degrees of development in managerial terms, even though each region acts as a public holding and applies the same external constraints to all PHOs. For this exploratory qualitative research, we used a multiple case study approach [15] to study three LHAs in the same region. The use of case studies was intended to minimise the influence of extraneous factors. Crucial for the present analysis, LHAs operate in similar environments that tend not to be influenced by differences in the local population.

We selected the Emilia-Romagna region in central Italy (population 4.4 million; capital Bologna), because it is one of the most innovative Italian regions from a managerial point of view [16]. Emilia-Romagna was one of the first regions to introduce a cost accounting system into its LHAs [17,18], and has issued precise instructions on strategic planning in its PHOs, in terms of both legislation and governance.

We selected three LHAs for the present study, namely Parma, Reggio Emilia, and Cesena. Since the early 1990s, these three LHAs have distinguished themselves by their ability to introduce managerial innovations [19]. Furthermore, the selected LHAs are all members of a benchmarking network (managed by Bocconi University, Milan) that connects 30 LHAs within the SSN. The scope of the topics discussed by the network includes strategic planning and modes, and processes of control. The participation of the LHAs in the network allowed us to work closely with them, which facilitated our access to the relevant strategic documents. The characteristics of the three selected LHAs are shown in Table 1.

**Table 1:** Characteristics of the selected cases (data shown from 2009)

**LHA**	**Name**	**Population served**	**Annual budget (million Euros)**	**Employees**	**Number of independent hospital trusts**	**Number of GPs**
LHA 1	Cesena	194,000	428	2800	0	173
LHA 2	Reggio-Emilia	525,000	910	4100	1	410
LHA 3	Parma	437,000	791	2500	1	360

### Data analysis model

In order to analyse the number and types of formally adopted strategic documents (first research question), representatives of the study LHAs participated in an intensive two-day training seminar. This seminar introduced the definitions and theoretical frameworks discussed in Section 1, in order to begin the process of ‘naming’ and ‘framing’ the problem. Then we interviewed three top managers of each PHO in the sample, including one of the senior managers (general director, healthcare director, or administrative director), the chief controller, and an operations manager. Finally, we jointly selected the most relevant strategic planning documents, which had been formally adopted between 2004 and 2010 in each PHO.

Second, we assessed the selected strategic documents in order to evaluate their principal focus. The following five planning focuses were considered: (1) the vision statement, (2) the planned service mix and portfolio evolution, (3) future resource allocation, (4) planned organisational developments, and (5) accountability content and modes to external stakeholders. Using content analysis, each document was analysed to determine the number of times that key words appeared in the text relative to the overall number of pages of the document, in order to create an index to capture the document’s focus. The indicators represent how frequently the key words of each focus appear in the whole document. The numerator is expressed as the total number of times that the key word linked to a specific focus was present in the document, while the denominator is expressed in terms of the total number of pages, in order to make documents with different lengths comparable. The index helped us to establish the focus, or focuses, of the document, where a high number associated with an indicator corresponded to a high relevance of that focus. Average frequency of relevant words per page for each focus was also considered to evaluate the relevance of a single focus within a document.

We also carried out an in-depth analysis by reading all documents and recording their details in a database. In order to control for classification errors during the analysis process, a number of rules of evaluation were agreed. The authors worked independently, and discrepancies in the entries of the researchers were discussed before agreement was reached. We then adopted the sequence of a rational planning framework, first defining the vision statement and planned strategic position, then selecting the service portfolio and resource allocation, and ending with organisational development and stakeholder accountability [7,20,21] in order to assess the degree of logical correlation between strategic documents adopted in consecutive time periods. We also assessed the legislative framework in order to establish an ideal model of hierarchy and the logical correlations between strategic documents (see Table 2).

**Table 2:** Hierarchical and chronological coherence among strategic documents: the ideal model

**2007**	**2008**	**2009**	**2010**	**2011**	**2012**
	STRATEGIC VISION				
		PORTFOLIO OF ACTIVITIES			
		RESOURCE ALLOCATION			
		ORGANISATIONAL DEVELOPMENT			
			ACCOUNTABILITY		

The ideal model was built given the legislative framework and a rational strategic planning approach. Horizontal lines represent the validity in time of the documents.

The adoption date and planned implementation period of each document and its focus allowed us to observe the logical interactions between the different planning stages. We represented these interconnections graphically in order to capture an overarching view of adoption times and strategic focuses, and compared them with the ideal model obtained from previous studies of rational planning and the legislative framework. The aim of this step was to evaluate the degree of coherence and coordination between different strategic documents (i.e., the second research question).

Finally, we tabulated the major goals of each strategic plan in order to evaluate their consistency, contradictions, and redundancies (i.e., the third research question). Our aim was to assess whether there was a general consistency and disclosure in terms of the objectives and the allocation of resources in the documents, and to understand the purpose of the strategic documents. For this reason, we selected four main targets from the first documents adopted by each PHO between 2004 and 2012, in order to assess how each goal was linked to the contents of the succeeding documents. We used five defined levels of consistency in order to evaluate, for each LHA, the overall consistency of goals among strategic documents. The aim of this step was to categorise each LHA in terms of level of consistency, based on the connection between documents. The five levels were defined as follows:

1. LEVEL 1: General goals are clearly stated and easily detectable in the first strategic document;

2. LEVEL 2: General goals are clearly stated and easily detectable in the first document; and corresponding variations in the level of services are detectable in the succeeding documents;

3. LEVEL 3: General goals are clearly stated and easily detectable in the first document; corresponding variations in the level of services are detectable in the succeeding strategic documents; and variations in the level of resources/investments are directly linked to the specific goal;

4. LEVEL 4: General goals are clearly stated and easily detectable in the first document; corresponding variations in the level of services are detectable in the succeeding documents; variations in the level of resources/investments are directly linked to the specific goal; and there is a clear link between all documents for each single specific goal;

5. LEVEL 5: General goals are clearly stated and easily detectable in the first document; corresponding variations in the level of services are detectable in the succeeding documents; variations in the level of resources/investments are directly linked to the specific goal; there is a clear link between all documents for each individual goal; and all documents are designed using similar language, layout, and articulation of goals.

All five levels are logically linked and the achievement of a higher level implies the attainment of the previous level. If the characteristics of a lower level are not met, it is not possible to reach a higher degree of overall consistency between documents.

## Results

### The number and types of strategic documents

From the data analysis, the first point of interest was the breadth of the strategic documents adopted by the three LHAs in the short study period. Generally, no single comprehensive strategic document exists. Instead, there is a series of plans defining similar items in different ways, proposing different content, focussing on different priorities, and adopted at different times. Each LHA selected more than one document that they defined to be ‘strategic’ (LHA 1 and LHA 2 selected six documents, while LHA 3 selected eight). This breadth of strategic documents differs from the findings of previous authors, who have tended to depict the strategic planning process as coordinated and linear, articulated in rational stages, and resulting in the creation of one overarching strategic document [7,20]. Furthermore, the selected planning documents had different types of content and different structures, indicating that LHAs (and PHOs in general) are overloaded with formal strategic planning documents. Thus, although the strategic culture of NPM has strongly influenced the Italian National Health Service by promoting the adoption of strategic planning documents, the huge number of ‘strategic’ documents can impede successful strategic management [22]. Strategic planning documents may have a bureaucratic origin, when they represent an administrative constrain imposed by law or by a higher level of Government. Otherwise they may represent an internal managerial resolution in order to steer the organization. External legal constrains do not impose logical links among all strategic documents, neither the attainment of specific overall goals. Each document explicitly pursues different objectives. The differences among them are the result of legislative stratifications or of fragmented powers in the public network.

### Focuses of the studied documents

The presence of multiple strategic documents, instead of an overarching strategic plan, can only be justified by the different focuses of each document. Using the content analysis approach described in Section 2, we clearly demonstrated that several documents had more than one focus and that these focuses are repeated in multiple documents for each LHA (Table 3). For instance, LHA1 had overlap regarding their strategic vision (documents 1, 2, 3, 5) and resource allocation (documents 4, 5, 6). LHA 2 and 3 had strategic planning documents mainly focused on strategic vision and organisational development. In general, these two focuses were the most frequent among all documents analysed (average frequency 0.94 and 1.46 per page). Moreover, four types of content (strategic vision, portfolio of activities, resource allocation, and organisational development) were repeated and one (accountability to stakeholders) was almost entirely absent (lowest frequency among all focuses: 0.31 per page). In fact, the index of frequency for this focus was in all cases (except for document 2 of LHA 2) lower than the average. External accountability to stakeholders might hardly have been considered because strategic documents themselves may be considered a means of accounting to external stakeholders. Indeed, because these strategic documents repeat the same topics, they presumably represent different means of ‘selling’ the content to diverse stakeholders. LHAs use different documents in their interactions with different stakeholder groups, thereby discussing similar topics using different language, formats and focuses.

**Table 3:** Focus of the documents and overall level of consistency and disclosure

		**Number of pages**	**Strategic vision**	**Portfolio of activities**	**Resource allocation**	**Organisational development**	**Accountability**	***Average***	**Level of consistency and disclosure among all documents (from 1 to 5)**
LHA 1	Document 1	70	0.37	0.04	0	0.42	0.01	*0.17*	3
	Document 2	88	1	0.14	0	0.35	0.18	*0.33*	
	Document 3	52	0.8	0.06	0.02	0.52	0.54	*0.39*	
	Document 4^1^	416	-	-	-	-	-	*-*	
	Document 5^1^	88	-	-	-	-	-	*-*	
	Document 6^2^	137	-	-	-	-	-	*-*	
LHA 2	Document 1	96	1.19	0.13	0.06	2.55	1.13	*1.01*	3
	Document 2	34	2.08	0.2	0.03	1.67	0.61	*0.92*	
	Document 3^2^	31	0.12	0.06	0.03	0.06	0.03	*0.06*	
	Document 4	104	1.8	0.35	0.01	0.75	0.29	*0.64*	
	Document 5	20	1.75	0.85	0	1.2	0.75	*0.91*	
	Document 6	175	0.77	0.07	0.02	0.24	0.05	*0.23*	
LHA 3	Document 1	51	0.76	0.45	0.14	2.58	0.8	*0.95*	2
	Document 2	104	0.78	0.8	0.02	0.07	0.13	*0.36*	
	Document 3	102	0.63	0.64	0	10.45	0.27	*2.40*	
	Document 4	205	0.94	0.33	0.02	0.66	0.13	*0.42*	
	Document 5	184	1.05	0.15	0	1.2	0.16	*0.51*	
	Document 6	74	0.51	0.14	0.01	0.12	0.06	*0.17*	
	Document 7	52	1.32	1.06	0.02	1.98	0.21	*0.92*	
	Document 8^2^	18	0.05	0	0	0	0	*0.01*	
*Average*			*0.94*	*0.32*	*0.38*	*1.46*	*0.31*		

^1^ Quantitative content analysis not available (scanned documents). A qualitative analysis was carried out.

^2^ Budget.

Legend: The indicators represent how frequently the relevant words of each focus appear in the whole document (in terms of words per page).

The level of consistency and disclosure is categorised on a scale from one (low) to five (high).

### Degree of integration and coordination

The documents and their sequence were partially determined by the legislative framework defined by the regional government of Emilia-Romagna. Given the sequencing of legislation, which suggests the use of a rational strategic approach [7,20], it might be expected that the hierarchy of the documents would reflect a logical succession, as represented in Table 2. Thus, the first adopted strategic documents should define the strategic vision for the LHA, identify what type of strategic orientation it should adopt for the future (collaboration or competition, key specialisation in a public healthcare network, and so on), and offer a general framework to the organisation. This initial document should include a mission statement, main areas of intervention, as well as identify funding sources. Within this framework, the organisation should then use other formal planning tools in order to identify its future portfolio of services and activities and, consequently, to define how to allocate its resources. Thereafter, the succeeding planning documents should concentrate on organisational and operational developments at the micro level. Finally, they should also focus on communication and accountability to stakeholders.

The present study analysed the logical correlations between the planning tools for each PHO and their coordination in terms of date of adoption and time sequence. Although adoption dates usually are clearly shown in public documents, some documents still required careful reading of their introductory paragraphs in order to understand the time horizons involved. In some cases, the date of approval was after the formal planning timeframe, highlighting the first source of incoherence. By analysing the chronological order and focuses of the documents, we aimed to establish whether there was a high degree of overall coherence and a clear time sequence, implying a rational approach to strategic planning and a desire for effective organisational guidelines.

As shown in Tables 4, 5, 6, however, and contrary to our expectations, none of the study LHAs showed a logical and chronological succession of documents and focuses (see Table 2). Most documents were adopted and were in use between 2008 and 2012, with the exception of the LHA 2, which has a document (document 4) still in force, even if it was originally adopted in 2005. The majority of strategic documents analysed have a three- or one-year validity. The planning process at these LHAs appears to be poorly coordinated, with a number of oversights and a complex and diverse distribution of content between documents. Although a logical sequence of formal planning tools was expected, in most cases planning was duplicated in different periods and documents, confirming the incoherence and redundancy. Some items barely existed or were absent altogether. Furthermore, where strategic documents had expired they were rarely replaced immediately with an updated version, and the originals were still considered to be in force by the organisations. Although each LHA seemed to discuss and formalise its plans coherently, the sequence of adoption was inconsistent. In public organisations, the formal approval of strategic plans can vary a lot, either being very close to the period of analysis and strategic thinking, or significantly distanced therefrom. Formal approval seems to depend upon political expediency and the cycles adopted by stakeholders, whereas the internal timeframe of strategic design is more closely related to organisational needs, which cannot always be formalised in the public environment. 

**Table 4:** Hierarchical and chronological coherence among strategic documents: LHA 1

**2004**	**2005**	**2006**	**2007**	**2008**	**2009**	**2010**	**2011**	**2012**
		Document 1 (adopted in 2007) - Strategic vision + Org. Development (3 years)						
					Document 2 - Strategic vision (3 years)			
					Document 3 - Strategic vision (3 years)			
						Document 4 - Portfolio of activities + Resource allocation (1 year)		
						Document 5 - Strategic vision + Resource allocation + Portfolio of activities (3 years)		
						Document 6 - Resource allocation (1 year)		

Horizontal lines represent the validity in time of the documents.

**Table 5:** Hierarchical and chronological coherence among strategic documents: LHA 2

**2004**	**2005**	**2006**	**2007**	**2008**	**2009**	**2010**	**2011**	**2012**
Document 4 - Strategic vision (3 years, but still in force)								
					Document 1 - Org. Development (4 years)			
					Document 2 - Strategic vision (3 years)			
						Document 3 - Resource allocation (3 years)		
						Document 5 - Strategic vision + Organisational development (3 years)		
					Document 6 - Strategic vision (1 years)			

Horizontal lines represent the validity in time of the documents.

**Table 6:** Hierarchical and chronological coherence among strategic documents: LHA 3

**2004**	**2005**	**2006**	**2007**	**2008**	**2009**	**2010**	**2011**	**2012**
				Document 1 - Org. Development (5 years)				
					Document 2 - Strategic vision + Portfolio of activities (1 year)			
						Document 3 - Org. Development (1 year)		
					Document 4 - Strategic vision (3 years)			
						Document 5 - Strategic vision + Org. Development (1 year)		
					Document 6 - Strategic vision (3 years)			
						Document 7 - Strategic vision + Portfolio of activities + Org. Development (3 years)		
				Document 8 - Resource allocation (3 years)				

Horizontal lines represent the validity in time of the documents.

There does seem to be general compliance with the Italian legislative frameworks in terms of the adoption of formal documents. From this perspective, the effect of NPM culture on LHAs is reconfirmed. With regard to the theoretical framework presented earlier, strategic planning documents seem to comply with the general rules that govern their adoption and are a means to relate and account to stakeholders.

### Degree of disclosure and consistency of goals

The analysis in Table 3 shows that LHA 1 and LHA 2 had a medium degree of consistency and disclosure (both level 3), indicating that some variations in the allocation of resources and investments are linked to the general goals, but they still seem to use different language. LHA 3 had an even lower degree of consistency and disclosure (level 2) because, having detected the main targets in the first strategic documents, in the succeeding ones we found variations in the mix and level of service provision, without any specification of how resources and investments vary. Among the eight documents selected for LHA 3, it was difficult to construct a path from the main goal to defined concrete actions in other documents with a different or a similar focus.

In general, it was difficult to find a strong overall consistency between documents, and impossible to determine how each general goal translated into concrete actions with a clear allocation of resources and funding. In most cases, the general objectives tended to be more specific in later documents; however, no explicit evidence of the available resources for a specific project or the types of investment was shown. Indeed, this analysis emphasises that, even in the presence of a logical correlation between documents, there is a significant lack of consistency, and goals are ambiguously presented.

In general, the content of the documents presents an incomplete picture of goals and targets. All the strategic plans featured a vast amount of historical data on the context and results achieved in the organisation concerned, followed by vague qualitative statements about future strategy, and missing or incomplete definitions of quantitative targets and resource allocations.

The only targets stated were improvements to particular services, usually without any quantification of the expected improvement. Moreover, these services are often peripheral activities. In addition, no reductions in services are ever described, and changes in the allocation of resources are usually unavailable. Although each LHA produces its own formal balance sheets, these are not embedded or summarised in its strategic documents. Indeed, the strategic documents seem to focus on the reconstruction and analysis of past performance and results, and on the promotion of a qualitative vision of service orientation and organisational development. However, relevant or systematic data on future strategy are rarely provided.

Public organisations rarely define clear targets, even in their formal plans, leading to uncertainty as to which social target has been prioritised and which have been partly or totally excluded [23]. However, admittedly, some opacity is necessary for the promotion of organisational development in order to meet the expectations of the professionals involved [23].

This problem of quantification is consistent with traditional Weberian bureaucracies, which engage in planning using an input-based approach in order to discern all the issues related to the financial statements, but hide information about user targets, included and excluded social clusters, outputs, service standards, and equity.

## Discussion

The aim of the current study was to analyse the role played by strategic documents in PHOs given their degree of overall coherence. Using three case-study LHAs, we analysed the logical correlations between the different strategic documents adopted by individual PHOs in order to understand their degree of coherence, coordination and disclosure, and the role they play in management steering the organisation and accounting to stakeholders.

The results show that NPM prescriptions had an impact on the PHOs in our sample, since there is a diffused strategic culture and planning tools are widespread. Formally adopted strategic plans are embedded and capture health sector complexity, trying to analyse and regulate a wide portfolio of issues and to dialogue with a rich arena of stakeholders.

There is a multitude of formal strategic documents in place, although they are often uncoordinated and have inconsistent content. There is often no logical sequence between strategic documents, even though all of them are consistent with different stakeholder needs and perspectives. Almost every strategic document has an external focus because of the bounds set by regional regulations or government policy, as well as by stakeholder expectations.

The strategic documents of LHAs are the main tools for communicating with diverse stakeholders that use different languages and varied processes of negotiation. As such, LHAs must use their influence to alter language, format, processes, and timeframes in order to meet stakeholder needs [24]. Although the content of these documents must be sufficiently coherent as befits a strategic design process, they must also be sufficiently differentiated in order to meet the expectations of stakeholders.

Officially adopted strategic plans offer vague and qualitative targets for steering complex PHOs. Although a rich diversity of quantitative reports is available, these reports are always related to past performance, and analytic and clearly stated future goals are not in place. Future strategies are described using only a qualitative and narrative approach [25]. Stakeholders are enabled to understand past performance and critical issues, while for the future the debate is more about strategic vision and qualitative choices.

## Conclusion

These considerations all belong to a complex and multi-actor model of social bargaining that focuses on sense-making and political representations rather than on quantitative planning [26]. PHOs are among the most pluralistic organisations [27], meaning that many external stakeholders and competitors apply severe pressures that affect the organisation’s strategy. On the other hand, internal pluralistic tensions are also typical features of professional organisations such as PHOs, where different cultures - medical and administrative - and various subcultures related to medical disciplines, legitimately participate in the formulation and implementation of a strategy with their specific interests and goals in mind [28]. Consequently, the powerful and conflicting interests of internal and external stakeholders slow down or prevent actors from finding a shared strategic consensus and defining precise quantitative targets. In other words, this discourages strategy from being an explicit, coherent and unified direction for the organisation as emphasized by the traditional theory. The resulting strategic ambiguity could produce a strategic paralysis or a dilution in strategic change initiatives [29]. This is strongly consistent with the results of our study, where we could not find a unified strategy explicit and coherent across all strategic documents of each LHA. Rather, we found different goals, and different language, with the aim of answering to different stakeholders’ expectations. Scholars confirm that public hospitals’ strategic plans are often heavily oriented towards a generic development and have ambiguous and loosely integrated goals [30].

Therefore, in highly pluralistic contexts, participatory strategy approaches are more effective than rational ones. Scholars agree that strategy-making in these organisations requires collaborative decision-making processes involving the plurality of actors playing distinct but tightly-knit roles [31].

In summary, no clear or explicit overarching quantitative strategy is available and all strategic planning documents seem to be externally oriented: they communicate similar topics to different stakeholders, each of whom use a different language, and have a different negotiation process and informational needs. From this point of view, strategic planning documents represent a means of gaining and developing a protected space for ‘hidden’ decision-making, which might be necessary in order to allow PHOs to take autonomous managerial decisions without the involvement of the external stakeholders who were involved in the formal process of drafting these documents. This is a consequence of the complexity of PHOs, and public managers in the healthcare sector should be aware of and not victim to this complexity. As a consequence, management should base strategic decision-making on an awareness of the different purposes of each planning document, assigning it the most appropriate role in the overall planning decision process. There can be many different purposes for these documents: communicating the organisation’s values, being accountable to internal or external stakeholders, presenting a long-term vision, planning the tools for managing the PHO. Documents’ titles are not always consistent with their content and the real meaning they have in managing the organisation and its relationship with the external context. Managers should use the various planning documents to govern the healthcare organisation and, at the same time, create favourable conditions for exchanges and consensus building with the external contexts and all stakeholders.

The evaluation of the extension of this strategic space, to be created by healthcare sector managers, and the explanation of how this can be effectively achieved through organisational autonomy without breaking stakeholder trust and social contracts, is a broad avenue for future research in this area.

## Abbreviations

LHA: Local Health Authority; NPM: New Public Management; PHO: Public Healthcare Organisation; SSN: Sistema Sanitario Nazionale (Italian National Health Service).

## Competing interests

The authors declare that they have no competing interests.

## Authors’ contributions

All authors contributed equally to this work.

## Pre-publication history

The pre-publication history for this paper can be accessed here:

http://www.biomedcentral.com/1472-6963/13/5/prepub
